# Epidemiology and distribution of 10 superantigens among invasive *Streptococcus pyogenes* disease in Germany from 2009 to 2014

**DOI:** 10.1371/journal.pone.0180757

**Published:** 2017-07-18

**Authors:** Matthias Imöhl, Christina Fitzner, Stephanie Perniciaro, Mark van der Linden

**Affiliations:** 1 Institute of Medical Microbiology and National Reference Center for Streptococci, University Hospital (RWTH), Aachen, Germany; 2 Department of Medical Statistics, University Hospital (RWTH), Aachen, Germany; Universidade de Lisboa Faculdade de Medicina, PORTUGAL

## Abstract

A nationwide laboratory-based surveillance study of invasive *S*. *pyogenes* infections was conducted in Germany. Invasive isolates (n = 719) were obtained between 2009 and 2014. Most isolates were obtained from blood (92.1%). The proportions of isolates from cerebrospinal fluid, pleural fluid, synovial fluid and peritoneal fluid were 3.9%, 1.8%, 1.7% and 0.6%, respectively. The most common *emm* types were *emm* 1 (31.8%), *emm* 28 (15.4%) and *emm* 89 (14.5%). The most common superantigen genes (*spe*A, *spe*C, *spe*G, *spe*H, *spe*I, *spe*J, *spe*K, *spe*L, *spe*M, *ssa*) identified from *S*. *pyogenes* were *spe*G (92.1%), *spe*J (50.9%), and *spe*C (42.0%). Significant associations of superantigen genes with underlying conditions or risks were observed in *spe*G, *spe*H, *spe*J, and *spe*K. Significant associations between *emm* types or superantigen genes with clinical complications were observed in *emm* type 3 and in superantigen gene *spe*A 1–3. Most frequent clinical manifestations included sepsis 59.4%, STSS 6.3%, meningitis 5.4%, and necrotizing fasciitis 5.0% (significantly associated with *emm1*).

## Introduction

*Streptococcus pyogenes* (Lancefield group A streptococcus; GAS) is a major human pathogen and responsible for a wide range of both suppurative and non-suppurative diseases, e.g. pharyngitis, erysipelas, septicaemia, meningitis, pneumonia and the notably severe manifestations necrotising fasciitis (NF) and streptococcal toxic shock syndrome (STSS). Suppurative infections and post-infection sequelae, e.g. acute rheumatic fever, rheumatic heart disease and glomerulonephritis, result in substantial human morbidity [1]. Invasive infections caused by *S*. *pyogenes* (iGAS) have been reported increasingly since the mid- to late 1980s [2], and recent upsurges in iGAS infections were reported from England [3], Ireland [4, 5] and Sweden [6]. The global burden of invasive *S*. *pyogenes* disease is high, and there are estimated to be at least 663,000 new cases and 163,000 deaths worldwide each year. Beyond this, there are more than 111 million cases of *S*. *pyogenes* pyoderma and over 616 million cases of pharyngitis annually [7]. Among the many virulence factors produced by *S*. *pyogenes*, the M protein is considered to be of major importance. The M protein is a fimbrial protein located on the cell surface. The *emm* gene, which encodes the M protein, is used as the basis for typing *S*. *pyogenes*. Marked changes in the distribution of *emm* types circulating in Europe have been noticed over the last three decades [2]. Furthermore, there seem to be huge differences concerning the global distribution of *emm* types. A systematic review of the global distribution of GAS *emm* types found the epidemiology in Africa and the Pacific region to be different from that in other regions, particularly high-income countries. In Africa and the Pacific, there were no dominant *emm* types and a higher diversity of *emm* types, and many of the *emm* types common in other parts of the world were less common (including *emm* 1, 4, 6 and 12) [8].

In particular, *emm* 1, and, to a lesser extent, *emm* 3, are associated with outbreaks and fatal outcomes [2, 9–12]. A recent study analysing the epidemiological patterns of severe *S*. *pyogenes* disease in 11 European countries found an overall 7-day case fatality rate of 19%, ascending to 44% among patients who developed streptococcal toxic shock syndrome [13]. In comparison, these case fatality rates are lower than those reported in iGAS infections during a previous surveillance period in Germany (1996–2002: overall 40.6%, STSS 57.9%) [9]. Other important virulence factors include the streptococcal superantigens (SAgs). SAgs are bacterial toxins which bind to major histocompatibility complex class II and T-cell receptors, thereby stimulating large numbers of T cells and causing a massive release of cytokines into the bloodstream. Overproduction of these cytokines can lead to tissue damage, organ failure, and shock [14]. Currently, eleven different superantigens (*spe*A, *spe*C, *spe*G, *spe*H, *spe*I, *spe*J, *spe*K, *spe*L, *spe*M, *ssa*, *sme*Z) have been identified from *S*. *pyogenes* [14–18].

In this study, we analysed the superantigens *spe*A, *spe*C, *spe*G, *spe*H, *spe*I, *spe*J, *spe*K, *spe*L, *spe*M, and *ssa* for all isolates. The present investigation compares the *emm* types and the superantigen toxin genes of 719 invasive *S*. *pyogenes* strains collected in a nationwide voluntary laboratory-based surveillance in Germany during 2009 to 2014. Clinical manifestations, clinical complications, underlying conditions and risk factors are analysed.

## Materials and methods

### Study design

German microbiological laboratories were invited to send their isolates to the German National Reference Center for Streptococci (NRCS; Aachen, Germany). In total, 719 isolates were sent by 130 laboratories located all over Germany between 2009 and 2014. Isolates were included into the study when they met the criteria of an invasive infection according to the definition of the Working Group on Severe Streptococcal Infections 1993 [19], i.e. isolation from a normally sterile site (e.g., blood, cerebrospinal fluid, synovial fluid).

In order to collect the underlying data, a detailed questionnaire was filled out for each specimen sent by the participating centers. In the few cases without enclosed questionnaires, the completion of the data sheet was requested retrospectively. The data included gender and age of the patient, the diagnoses (including certain specified diagnoses like STSS, NF, septicaemia, pneumonia, cellulitis and puerperal sepsis), and information about the clinical course (including information about presence of shock, adult respiratory distress syndrome, presence of artificial ventilation, renal failure, soft-tissue necrosis, disseminated intravascular coagulation, liver abnormality and exanthema). Possible risk factors analysed included the presence of immunosuppression, concomitant surgery, diabetes mellitus, chronic skin lesions, hospital acquired infection and intravenous drug abuse.

### Microbiological investigations

Isolates were identified by *β*-haemolysis on sheep blood agar, Lancefield antigen grouping using a commercially available agglutination technique (Slidex Streptokit, bioMérieux, Marcy-L’Etoile, France; Prolex Streptococcal Grouping Latex Kits, Pro-Lab Diagnostics, Richmond Hill, Canada), and the pyrrolidonyl-arylamidase (PYR) test. The detection of *emm* genes was determined by PCR using ‘all M primers’ as described previously [20]. PCR products were purified and sequenced using an automated ABI Prism 310 DNA sequencer (Applied Biosystems, Weiterstadt, Germany). The nucleotide sequences encoding the N-terminal hyper-variable portion of the M protein were compared to the *emm* database and *emm* types were assigned as described on the CDC’s website (http://www2a.cdc.gov/ncidod/biotech/strepblast.asp). The presence of the ten different superantigen genes (*spe*A, *spe*C, *spe*G, *spe*H, *spe*I, *spe*J, *spe*K, *spe*L, *spe*M, *ssa*) was determined by PCR as described previously [16]. Concerning *spe*A, the primer spea1-4 detects the spe*A* alleles 1, 2, 3, and 4, whereas the primer spea1-3+5 detects the spe*A* alleles 1, 2, 3, and 5. If spea1-4 and spea1-3+5 both yield positive results, then the isolate contains *spe*A allele 1, 2, or 3. If only spea1-4 gives positive results, then the isolate contains the allele spe*A* 4; if only spea1-3+5 gives positive results, then the isolate contains the allele spe*A* 5 [14].

### Statistical analysis

Continuous variables were summarized by means and corresponding standard deviations. Categorical variables were summarized by absolute and relative frequencies. Univariate logistic regression models were used for variable selection, and a selection criterion of p < 0.05 was used for inclusion into multivariate logistic regression models. For each *emm* type and each superantigen, we investigated the possible influence on each outcome parameter (all diagnoses and all clinical complications). In contrast, the influence of all risk factors was investigated for all *emm* types and all superantigens (here the outcome parameter). Influence factors with a p value of p < 0.05 as well as age and sex were selected for the corresponding multivariate models. Univariate models were also constructed to examine possible relationships between *emm* type and superantigen genes. For sex, odds ratios >1 correspond to relationships which occur more commonly in males, while odds ratios <1 occur more commonly in females. For age, odds ratios >1 correspond to a relationship with increasing age, and odds ratios <1 correspond to decreasing age. Only outcome variables with 15 or more events are analysed in regression models [21]. Further possible estimation problems are described in the discussion section. All tests were two-sided and assessed at the 5% significance level. Because of the exploratory nature of the study we made no adjustment to the significance level of the several multivariate models. Statistical analyses were performed using R software, version 3.3.2. The complete data set used for the analyses is included as a supplementary table, S1 Table.

### Ethical statement

An ethical approval or patients’ consent was not required since the study only includes microbiological samples sent to the German National Reference Center for Streptococci on an anonymized basis by the sending microbiological laboratories, and did not involve human subjects or material.

## Results

**Fig 1:**
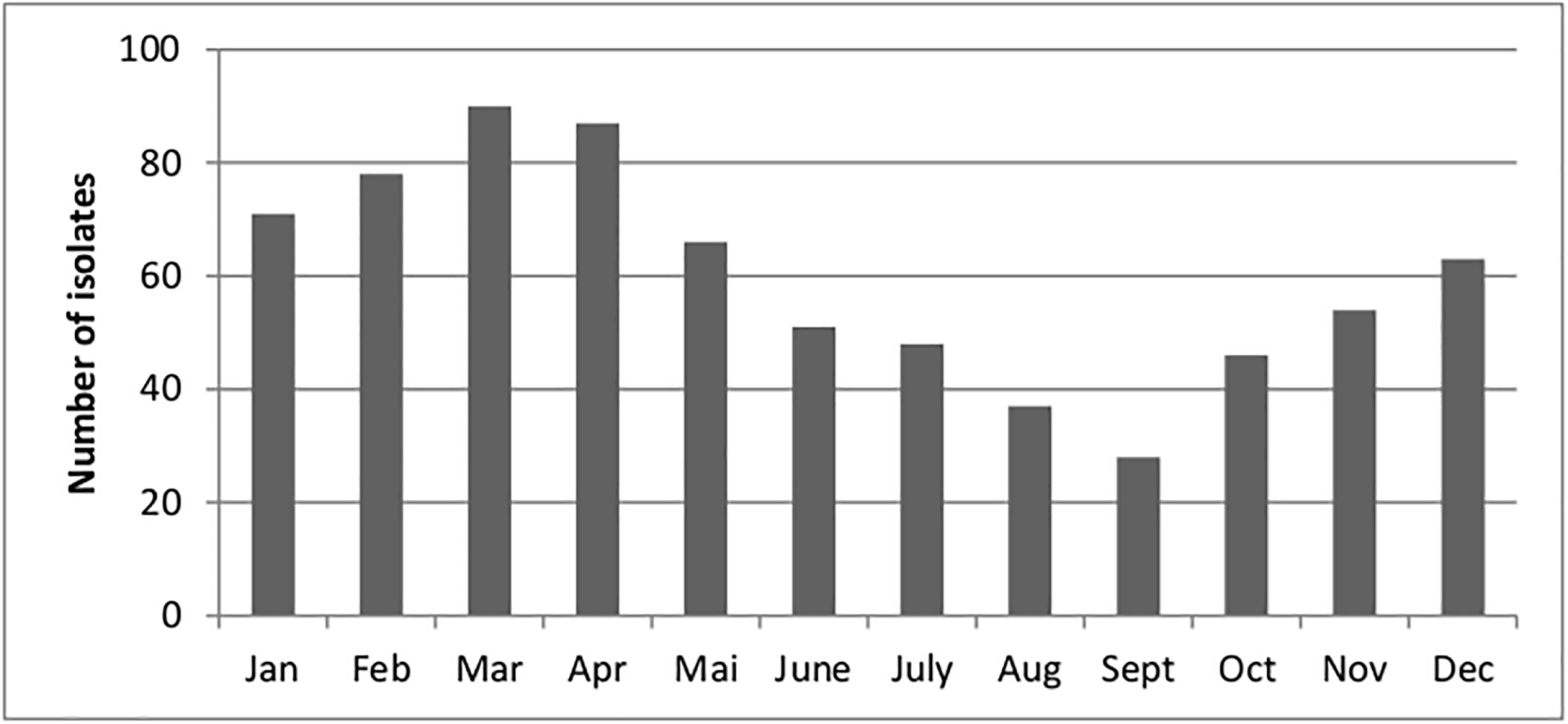
Seasonal distribution of 719 iGAS cases in Germany (2009–2014).

A total of 719 iGAS samples were collected between January 1st 2009 and December 31st 2014. The numbers of included cases for each year were: 2009, 91; 2010, 112; 2011, 108; 2012, 122; 2013, 155 and 2014, 131. The isolates were obtained from blood (662), cerebrospinal fluid (28), pleural fluid (13), synovial fluid (12), and peritoneal fluid (4). A seasonal variation was noted, with most cases reported in winter and early spring (**Fig 1**).

**Fig 2:**
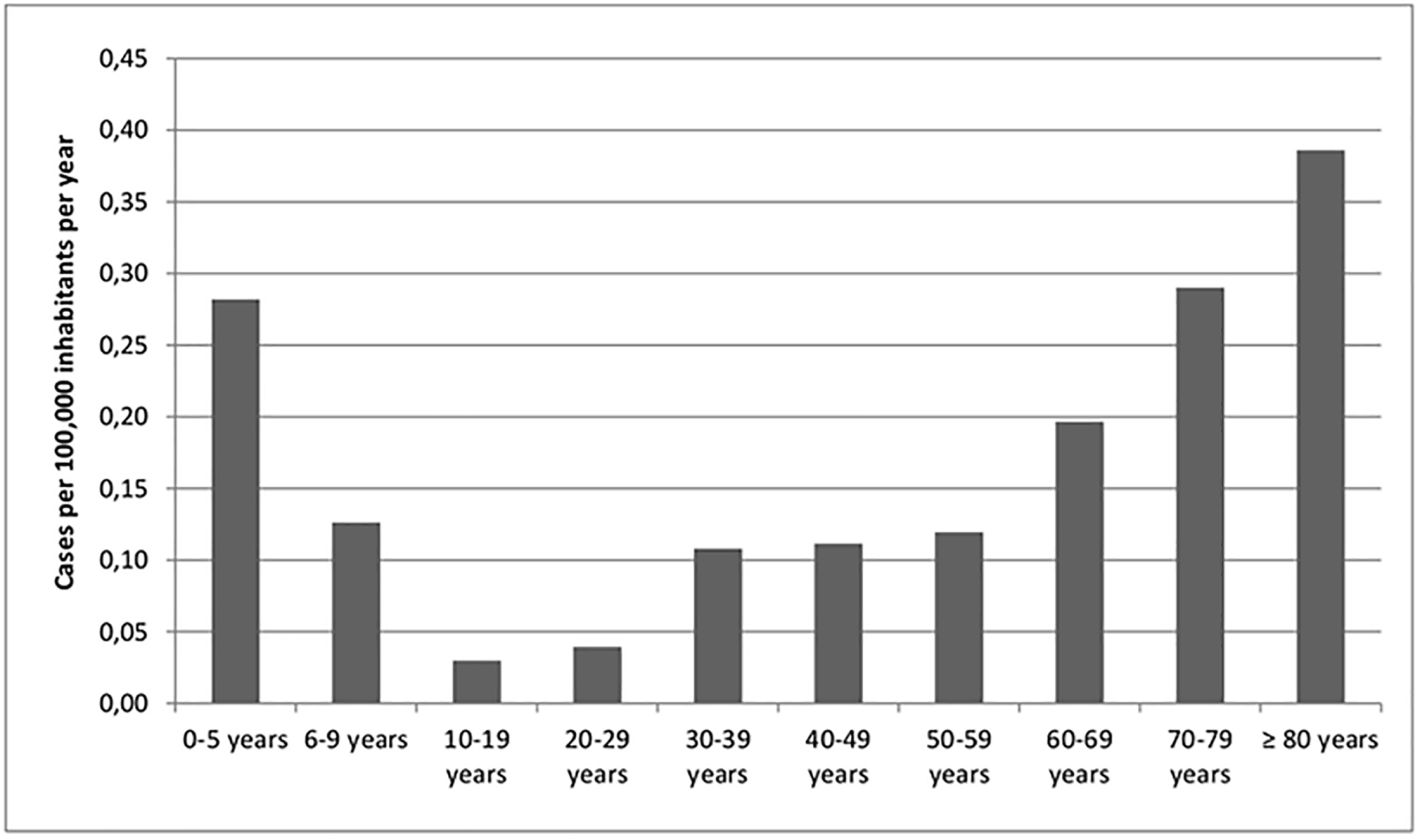
Age distribution of iGAS disease in Germany per 100,000 inhabitants per age group per year from 2009 to 2014 (n = 719) based on a population of 80,767,463 in 2013 (http://www.destatis.de).

The age-specific incidence of iGAS infections is shown in **Fig 2**. For the 719 patients, the mean age was 53.5 years, the median 59 years (range 0–97 years). A higher amount of cases per 100,000 inhabitants in the respective age groups was found in children up to 5 years and in adults ≥ 60 years. Among the latter, in our study the incidence was relatively constant among adults in the age groups from 30 to 59 years, but rose with every decade of age among those aged ≥ 60 years. For adults from 70–79 years, and especially those aged ≥ 80 years, the incidence even exceeded those among children from 0–5 years of age.

Most isolates (53.1%) were obtained from male patients, 46.7% from females and for 0.1% no information on gender was available. All patient data are listed in S1 Table.

Information on the existence of underlying conditions/risk factors was available for 95 (13.2%) of the 719 cases; 2.5% of these patients had two, and 0.3% had three risk factors. Most frequent risk factors were diabetes (43.2%), immunosuppression (29.5%) and chronic skin lesions/wounds (24.2%) (**Table 1**). The distribution of clinical manifestations and clinical complications of iGAS is shown in **Table 1** as well. The most common clinical manifestations were sepsis (59.4%), followed by erysipelas (7.6%), pneumonia (7.0%), STSS (6.3%), and meningitis (5.4%). Next frequent manifestations were NF (5.0%), phlegmon (4.0%) and septic arthritis (2.1%). Four cases of puerperal sepsis (0.6%) were reported from 2009 to 2014. Among the clinical complications of iGAS infections, hypotensive shock (17.8%) was the most common condition, followed by renal insufficiency (15.7%), disseminated intravascular coagulopathy (DIC) (11.8%), liver abnormality (10.2%) soft tissue necrosis (8.6%), and respiratory distress (6.5%).

**Table 1:** Underlying conditions, diagnoses and clinical complications among iGAS cases in Germany (2009–2014).

	No. of Cases	Percentage %
Underlying condition*		
Diabetes	41	43.2
Immunosuppression	28	29.5
Chronic skin lesions/Wound	23	24.2
Surgical operation within 7 days	13	13.7
Current injection drug use	6	6.3
Hospital acquired infection	5	5.3
NSAID^a^	1	1.1
**Diagnosis**		
Sepsis	427	59.4
Erysipelas	55	7.6
Pneumonia	50	7.0
STSS	45	6.3
Meningitis	39	5.4
Necrotizing fasciitis	36	5.0
Phlegmon	29	4.0
Septic arthritis	15	2.1
Endocarditis	8	1.1
Pleural empyema	5	0.7
Puerperal sepsis	4	0.6
Peritonitis	4	0.6
Osteomyelitis	2	0.3
**Clinical complications**		
Hypotensive shock	128	17.8
Renal insufficiency	113	15.7
DIC ^b^	85	11.8
Liver abnormality	73	10.2
Soft tissue necrosis	62	8.6
Respiratory distress	47	6.5
Exanthema	17	2.4

* Information about the underlying conditions was available only for 95 (13.2%) of the 719 cases. Percentages in this section of the table refer to these 95 isolates. 75 cases had one underlying condition, 18 cases had two underlying conditions, and two cases had three underlying conditions.

^a^ NSAID, nonsteroidal anti-inflammatory drugs.

^b^ DIC, disseminated intravascular coagulopathy.

Among the 719 isolates, 46 different *emm* types were identified (**Table 2**). The five most common types, *emm* 1 (31.8%), *emm* 28 (15.4%), *emm* 89 (14.5%), *emm* 3 (7.9%), and *emm* 12 (6.4%), are responsible for 76.1% of iGAS disease. The frequency of *emm* type 28 isolates was fairly constant from 2009 to 2014, whereas the four other most prominent *emm* types (1, 89, 3, and 12) were more variable, though yearly variations in *emm* types did not show any significant patterns (see **Table 3**). 92.1% of samples were positive for *spe*G, 50.9% for *spe*J, 42.0% for *spe*C, 39.9% for *spe*A 1–3, no samples were positive for *spe*A 4, 0.7% for *spe*A 5, 14.3% for *ssa*, 14.5% for *spe*K, 9.7% for *spe*H, 7.4% for *spe*I, 5.7% for *spe*M, and 4.7% for *spe*L.

**Table 2:** Distribution of *emm* types and superantigen/toxin genes among the 719 iGAS-cases in Germany (2009–2014). Bold print indicates a statistically-significant positive association from univariate logistic regression analysis (p≤0.05) between the listed *emm* type and superantigen gene(s).

emm type	n	%	*spe*C	%	ssa	%	*spe*A1-3	%	*spe*A5	%	*spe*G	%	*spe* H	%	*spe*I	%	*spe*J	%	*spe*K	%	*spe*L	%	*spe*M	%	none	%
**1**	229	31.8	13	5.7	3	1.3	**226**	**98.7**	0	0.0	**226**	**98.7**	1	0.4	0	0.0	**223**	**97.4**	3	1.3	0	0.0	2	0.9	0	0.0
**2**	5	0.7	2	40.0	0	0.0	0	0.0	0	0.0	5	100	0	0.0	0	0.0	0	0.0	0	0.0	4	80.0	4	80.0	0	0.0
**3**	57	7.9	0	0.0	**52**	**91.2**	**36**	**63.2**	0	0.0	56	98.2	0	0.0	0	0.0	1	1.8	**48**	**84.2**	1	1.8	4	7.0	1	1.8
**4**	29	4.0	**27**	**93.1**	**28**	**96.6**	1	3.4	0	0.0	1	3.4	1	3.4	0	0.0	1	3.4	0	0.0	0	0.0	1	3.4	0	0.0
**5**	10	1.4	10	100	0	0.0	1	10.0	0	0.0	10	100	0	0.0	0	0.0	1	10.0	0	0.0	0	0.0	0	0.0	0	0.0
**6**	12	1.7	12	100	0	0.0	12	100	0	0.0	12	100	9	75.0	9	75.0	0	0.0	11	91.7	0	0.0	0	0.0	0	0.0
**9**	3	0.4	2	66.7	1	33.3	0	0.0	1	33.3	3	100	0	0.0	0	0.0	0	0.0	0	0.0	1	33.3	1	33.3	0	0.0
**11**	5	0.7	5	100	0	0.0	2	40.0	0	0.0	5	100	1	20.0	1	20.0	0	0.0	0	0.0	0	0.0	0	0.0	0	0.0
**12**	46	6.4	**29**	**63.0**	4	8.7	0	0.0	0	0.0	**46**	**100**	**46**	**100**	**38**	**82.6**	0	0.0	0	0.0	0	0.0	0	0.0	0	0.0
**18**	4	0.6	4	100	0	0.0	4	100	0	0.0	4	100	0	0.0	0	0.0	0	0.0	0	0.0	4	100	4	100	0	0.0
**22**	3	0.4	1	33.3	1	33.3	0	0.0	0	0.0	3	100	0	0.0	0	0.0	0	0.0	0	0.0	0	0.0	0	0.0	0	0.0
**27**	1	0.1	0	0.0	0	0.0	0	0.0	0	0.0	1	100	0	0.0	0	0.0	0	0.0	0	0.0	0	0.0	0	0.0	0	0.0
**28**	111	15.4	**105**	**94.6**	1	0.9	2	1.8	0	0.0	**109**	**98.2**	0	0.0	0	0.0	**109**	**98.2**	**23**	**20.7**	2	1.8	3	2.7	0	0.0
**29**	1	0.1	0	0.0	0	0.0	0	0.0	0	0.0	1	100	0	0.0	0	0.0	0	0.0	0	0.0	1	100	1	100	0	0.0
**32**	1	0.1	1	100	0	0.0	0	0.0	0	0.0	1	100	0	0.0	0	0.0	0	0.0	0	0.0	0	0.0	0	0.0	0	0.0
**43**	2	0.3	1	50.0	0	0.0	0	0.0	0	0.0	2	100	0	0.0	0	0.0	0	0.0	1	50.0	0	0.0	0	0.0	0	0.0
**44**	3	0.4	1	33.3	2	66.7	0	0.0	0	0.0	3	100	1	33.3	0	0.0	3	100	0	0.0	0	0.0	0	0.0	0	0.0
**58**	2	0.3	1	50.0	1	50.0	0	0.0	0	0.0	2	100	0	0.0	0	0.0	0	0.0	0	0.0	0	0.0	1	50.0	0	0.0
**59**	7	1.0	1	14.3	0	0.0	0	0.0	0	0.0	7	100	0	0.0	0	0.0	7	100	0	0.0	1	14.3	1	14.3	0	0.0
**60**	2	0.3	0	0.0	0	0.0	0	0.0	0	0.0	0	0.0	0	0.0	0	0.0	0	0.0	0	0.0	0	0.0	0	0.0	2	100
**63**	2	0.3	0	0.0	0	0.0	0	0.0	0	0.0	1	50.0	0	0.0	0	0.0	0	0.0	0	0.0	0	0.0	0	0.0	1	50.0
**65**	1	0.1	1	100	0	0.0	0	0.0	0	0.0	1	100	1	100	1	100	0	0.0	0	0.0	0	0.0	0	0.0	0	0.0
**66**	10	1.4	0	0.0	0	0.0	0	0.0	0	0.0	10	100	0	0.0	0	0.0	0	0.0	0	0.0	0	0.0	0	0.0	0	0.0
**73**	1	0.1	1	100	0	0.0	0	0.0	0	0.0	1	100	0	0.0	0	0.0	1	100	0	0.0	0	0.0	0	0.0	0	0.0
**75**	11	1.5	2	18.2	0	0.0	0	0.0	2	18.2	11	100	0	0.0	0	0.0	0	0.0	1	9.1	11	100	11	100	0	0.0
**76**	4	0.6	1	25.0	0	0.0	0	0.0	0	0.0	4	100	0	0.0	0	0.0	3	75.0	0	0.0	0	0.0	0	0.0	0	0.0
**77**	19	2.6	12	63.2	0	0.0	0	0.0	0	0.0	1	5.3	1	5.3	1	5.3	0	0.0	1	5.3	1	5.3	1	5.3	5	26.3
**78**	1	0.1	1	100	0	0.0	0	0.0	0	0.0	1	100	0	0.0	0	0.0	0	0.0	0	0.0	0	0.0	0	0.0	0	0.0
**81**	5	0.7	1	20.0	0	0.0	0	0.0	0	0.0	5	100	2	40.0	0	0.0	2	40.0	0	0.0	0	0.0	0	0.0	0	0.0
**83**	2	0.3	0	0.0	0	0.0	0	0.0	1	50.0	2	100	0	0.0	0	0.0	0	0.0	0	0.0	2	100	2	100	0	0.0
**84**	1	0.1	0	0.0	0	0.0	0	0.0	0	0.0	1	100	1	100	0	0.0	1	100	0	0.0	0	0.0	0	0.0	0	0.0
**85**	2	0.3	0	0.0	0	0.0	0	0.0	0	0.0	2	100	0	0.0	0	0.0	2	100	0	0.0	0	0.0	0	0.0	0	0.0
**87**	7	1.0	5	71.4	5	71.4	1	14.3	0	0.0	7	100	1	14.3	0	0.0	7	100	0	0.0	0	0.0	0	0.0	0	0.0
**89**	104	14.5	**62**	**59.6**	1	1.0	0	0.0	0	0.0	**103**	**99.0**	1	1.0	0	0.0	0	0.0	15	14.4	4	3.8	3	2.9	0	0.0
**91**	2	0.3	0	0.0	0	0.0	0	0.0	0	0.0	2	100	2	100	2	100	2	100	0	0.0	0	0.0	0	0.0	0	0.0
**93**	1	0.1	0	0.0	0	0.0	0	0.0	0	0.0	1	100	0	0.0	0	0.0	0	0.0	0	0.0	1	100	1	100	0	0.0
**98**	1	0.1	0	0.0	0	0.0	0	0.0	0	0.0	1	100	0	0.0	0	0.0	0	0.0	0	0.0	0	0.0	0	0.0	0	0.0
**102**	1	0.1	0	0.0	1	100	0	0.0	0	0.0	1	100	1	100	1	100	0	0.0	0	0.0	0	0.0	0	0.0	0	0.0
**104**	1	0.1	0	0.0	0	0.0	0	0.0	0	0.0	1	100	0	0.0	0	0.0	0	0.0	0	0.0	0	0.0	0	0.0	0	0.0
**106**	1	0.1	0	0.0	1	100	1	100	0	0.0	1	100	0	0.0	0	0.0	0	0.0	0	0.0	0	0.0	0	0.0	0	0.0
**108**	2	0.3	0	0.0	2	100	0	0.0	0	0.0	2	100	0	0.0	0	0.0	2	100	0	0.0	0	0.0	0	0.0	0	0.0
**118**	3	0.4	1	33.3	0	0.0	0	0.0	0	0.0	3	100	0	0.0	0	0.0	0	0.0	0	0.0	0	0.0	0	0.0	0	0.0
**122**	1	0.1	0	0.0	0	0.0	0	0.0	0	0.0	1	100	1	100	0	0.0	0	0.0	1	100	0	0.0	0	0.0	0	0.0
**165**	1	0.1	0	0.0	0	0.0	0	0.0	0	0.0	0	0.0	0	0.0	0	0.0	0	0.0	0	0.0	0	0.0	0	0.0	1	100
NT*	1	0.1	0	0.0	0	0.0	0	0.0	1	100	1	100	0	0.0	0	0.0	0	0.0	0	0.0	1	100	1	100	0	0.0
**st854**	1	0.1	0	0.0	0	0.0	1	100	0	0.0	1	100	0	0.0	0	0.0	1	100	0	0.0	0	0.0	0	0.0	0	0.0
	719		302		103		287		5		662		70		53		366		104		34		41		10	

*NT = not typable

**Table 3:** Yearly distribution of *emm* types in 719 iGAS isolates from Germany.

	2009		2010		2011		2012		2013		2014		Total	
*emm* type	n	%	n	%	n	%	n	%	n	%	n	%	n	%
*emm*1	35	38.5	31	27.7	25	23.1	36	29.5	63	40.6	39	29.8	229	31.8
*emm*28	16	17.6	17	15.2	17	15.7	17	13.9	22	14.2	22	16.8	111	15.4
*emm*89	14	15.4	23	20.5	18	16.7	22	18.0	10	6.5	17	13.0	104	14.5
*emm*3	6	6.6	7	6.3	9	8.3	5	4.1	16	10.3	14	10.7	57	7.9
*emm*12	2	2.2	12	10.7	9	8.3	8	6.6	9	5.8	6	4.6	46	6.4
*emm*4	3	3.3	6	5.4	5	4.6	5	4.1	4	2.6	6	4.6	29	4.0
*emm*77	3	3.3	0	0.0	5	4.6	3	2.5	3	1.9	5	3.8	19	2.6
*emm*6	0	0.0	1	0.9	6	5.6	2	1.6	2	1.3	1	0.8	12	1.7
*emm*75	1	1.1	2	1.8	1	0.9	1	0.8	5	3.2	1	0.8	11	1.5
*emm*5	0	0.0	1	0.9	1	0.9	2	1.6	6	3.9	0	0.0	10	1.4
*emm*66	1	1.1	0	0.0	0	0.0	4	3.3	1	0.6	4	3.1	10	1.4
*emm*87	1	1.1	2	1.8	1	0.9	1	0.8	0	0.0	2	1.5	7	1.0
*emm*59	2	2.2	2	1.8	2	1.9	0	0.0	1	0.6	0	0.0	7	1.0
*emm*2	0	0.0	1	0.9	1	0.9	2	1.6	0	0.0	1	0.8	5	0.7
*emm*81	0	0.0	0	0.0	0	0.0	1	0.8	3	1.9	1	0.8	5	0.7
*emm*11	0	0.0	1	0.9	0	0.0	2	1.6	2	1.3	0	0.0	5	0.7
others*	7	7.7	6	5.4	8	7.4	11	9.0	8	5.2	12	9.2	52	7.2
total	91	100.0	112	100.0	108	100.0	122	100.0	155	100.0	131	100.0	719	100.0

* = others are *emm* types that occurred <5 times during the study period: *emm* 9, *emm* 18, *emm* 22, *emm* 27, *emm* 29, *emm* 32, *emm* 43, *emm* 44, *emm* 58, *emm* 60, *emm* 63, *emm* 65, *emm* 73, *emm* 76, *emm* 78, *emm* 83, *emm* 84, *emm* 85, *emm* 91, *emm* 93, *emm* 98, *emm* 102, *emm* 104, *emm* 106, *emm* 108, *emm* 118, *emm* 122, *emm* 165, NT.

The correlations between risks/underlying conditions, diagnoses and clinical complications and *emm* types or superantigens found in the statistical analysis are shown in **Tables 4, 5** and **6**. Among underlying conditions and risk factors, *spe*H, *spe*J, and *spe*K were significantly associated with chronic skin lesions, and *spe*G was significantly associated with diabetes. Among clinical complications, *emm* 1 was non-significantly associated with hypotensive shock, DIC, renal insufficiency, liver abnormality, soft tissue necrosis, and exanthema. Hypotensive shock, DIC, and renal insufficiency were non-significantly associated with *emm* 28. Superantigen *spe*C was non-significantly associated with DIC, renal insufficiency, and exanthema; *spe*A 1–3 with hypotensive shock, DIC, renal insufficiency, liver abnormality (significantly), soft tissue necrosis, and exanthema. Superantigen *spe*J was non-significantly associated with hypotensive shock and exanthema; *spe*M was non-significantly associated with soft tissue necrosis. In meningitis cases, *emm* types 1 and 89, as well as *spe*A 1–3 were predictors. *emm* 1, *spe*C, and *spe*J were all non-significantly associated with NF (*emm*1 reached statistical significance for NF) and sepsis. Septic arthritis was significantly associated with *emm* 28.

**Table 4:** Multivariate logistic regression results for underlying conditions and risk factors among iGAS cases with reported underlying conditions in Germany (2009–2014). Bold values indicate a statistically significant association in multivariate analysis.

*emm* type or superantigen	predicting factor	Odds ratio	95% CI		p-value
*spe*G	diabetes	0.9119	0.8366	0.9940	**0.0363**
	sex	0.9954	0.9567	1.0357	0.8211
	age in years	0.9997	0.9990	1.0005	0.4935
*spe*H	chronic skin lesions	1.1472	1.0147	1.2969	**0.0286**
	sex	1.0347	0.9910	1.0805	0.1220
	age in years	0.9987	0.9979	0.9996	**0.0028**
*spe*J	chronic skin lesions	0.7906	0.6428	0.9724	**0.0264**
	sex	0.9300	0.8646	1.0004	0.0517
	age in years	0.9985	0.9971	0.9999	**0.0355**
*spe*K	chronic skin lesions	1.1635	1.0053	1.3467	**0.0426**
	sex	1.0064	0.9559	1.0596	0.8080
	age in years	1.0011	1.0001	1.0021	**0.0284**

**Table 5:** Multivariate logistic regression results for clinical complications among 719 iGAS cases in Germany (2009–2014). Bold values indicate a statistically significant association in multivariate analysis.

Complication	predicting factor	Odds ratio	95% CI		p-value
hypotensive shock	*emm*1	1.1319	0.9740	1.3153	0.1065
	*emm* 28	0.9448	0.8268	1.0796	0.4042
	*spe*A 1–3	1.0008	0.9058	1.1058	0.9868
	*spe*J	0.9942	0.8830	1.1195	0.9238
	sex	0.9570	0.9051	1.0120	0.1234
	age in years	0.9997	0.9986	1.0007	0.5318
coagulopathy (DIC)	*emm* 1	1.0857	0.9937	1.1863	0.0692
	*emm* 28	0.9358	0.8696	1.0070	0.0766
	*spe*C	1.0366	0.9771	1.0997	0.2336
	*spe*A 1–3	1.0504	0.9652	1.1431	0.2547
	sex	0.9659	0.9216	1.0123	0.1482
	age in years	0.9988	0.9979	0.9997	**0.0089**
renal insufficiency	*emm* 1	1.0626	0.9609	1.1751	0.2373
	*emm* 28	0.9493	0.8734	1.0318	0.2213
	*spe*C	1.0021	0.9370	1.0717	0.9506
	*spe*A 1–3	1.0708	0.9727	1.1788	0.1632
	sex	1.0102	0.9578	1.0656	0.7086
	age in years	1.0011	1.0000	1.0021	**0.0414**
liver abnormality	*emm* 1	1.0004	0.9211	1.0864	0.9930
	*spe*A 1–3	1.0891	1.0072	1.1778	**0.0327**
	sex	0.9588	0.9176	1.0020	0.0617
	age in years	0.9996	0.9987	1.0004	0.3389
respiratory distress	*emm* 3	1.0900	1.0204	1.1644	**0.0106**
	sex	0.9723	0.9831	1.0077	0.1242
	age in years	0.9993	0.9986	0.9999	**0.0387**
soft tissue necrosis	*emm* 1	0.9926	0.9190	1.0720	0.8493
	*spe*A 1–3	1.0563	0.9822	1.1359	0.1402
	*spe*M	0.9217	0.8435	1.0071	0.0718
	sex	0.9859	0.9465	1.0271	0.4976
	age in years	1.0003	0.9996	1.0011	0.3883
Exanthema	*emm* 1	1.0137	0.9637	1.0663	0.5979
	*spe*C	1.0002	0.9735	1.0276	0.9903
	*spe*A 1–3	1.0285	0.9877	1.0709	0.1742
	*spe*J	1.0018	0.9724	1.0322	0.9043
	sex	0.9974	0.9754	1.0199	0.8196
	age in years	0.9995	0.9991	0.9999	**0.0329**

**Table 6:** Multivariate logistic regression results for diagnoses of 719 iGAS cases in Germany (2009–2014). Bold values indicate a statistically significant association in multivariate analysis.

Diagnosis	predicting factor	Odds ratio	95% CI		p-value
Meningitis	*emm* 1	1.0158	0.9557	1.0797	0.6147
	*emm* 89	0.9827	0.9357	1.0321	0.4869
	*spe*A 1–3	1.0368	0.9774	1.0998	0.2303
	sex	0.9791	0.9478	1.0115	0.2048
	age in years	0.9984	0.9978	0.9991	**0.000001**
Necrotizing Fasciitis	*emm* 1	1.0658	1.0117	1.1227	**0.0167**
	*spe*C	0.9885	0.9512	1.0272	0.5536
	*spe*J	0.9980	0.9563	1.0414	0.9249
	sex	0.9967	0.9653	1.0293	0.8421
	age in years	1.0001	0.9995	1.0007	0.8329
Septic Arthritis	*emm* 28	1.0528	1.0224	1.0841	**0.0006**
	sex	1.0056	0.9846	1.0270	0.6071
	age in years	1.0000	0.9996	1.0004	0.9079
Sepsis	*emm* 1	0.8515	0.7234	1.0022	0.0535
	*spe*C	0.9942	0.9113	1.0848	0.8966
	*spe*A 1–3	0.9849	0.8646	1.1219	0.8189
	*spe*J	1.0230	0.9292	1.1264	0.6430
	sex	1.0243	0.9532	1.1007	0.5132
	age in years	1.0012	0.9998	1.0026	0.0827

Additionally, we established some correlations between age, sex, and *emm* types and superantigen genes.Superantigen *spe*H was associated with decreasing age. Among clinical complications, coagulopathy, respiratory distress, and exanthema were significantly associated with decreasing age, while renal insufficiency was significantly associated with increasing age. Decreasing age was associated with *spe*H and *spe*J as predictors of chronic skin lesions. And in the studied diagnoses, meningitis was associated with decreasing age.

## Discussion

In this paper we present the results of 6 years of surveillance of iGAS disease in Germany.

Reported iGAS cases in Germany are low in comparison with surveillance programs from other countries. This might, at least in part, be explained by the voluntary nature of the German surveillance system, resulting in a smaller number of cases being referred to the reference laboratory and a potential underreporting of invasive *S*. *pyogenes* infections. In comparison with previous German surveillance periods, the incidence per year is slightly, but not significantly, higher in the current study (0.15 cases/100,000 individuals) than in previous surveillance periods (1996–2002, 0.1 cases [9]; 2003–2007, 0.13 cases [22]). The seasonal occurrence of iGAS disease with most cases reported in winter and early spring is congruent with the patterns observed in other countries [2].

In the present study, *emm* 1 was the most prevalent *emm* type, which is consistent with results from the USA [23], Australia [24], Japan [25], and across Europe [2, 9, 11], followed in frequency by the *emm* types 28, 89, 3 and 12. These five *emm* types are responsible for over three-fourths of iGAS disease in the current German surveillance period and these *emm* types have been reported to be among the most prevalent in the United States [23], Denmark [26, 27], and other European countries [11] as well. Compared to the two previous surveillance periods in Germany (1996–2002 [9] and 2003–2007 [22]), overall there seems to be no statistically-significant pattern in the frequency of *emm* type 1 (1996–2002, 37.3%; 2003–2007, 30.5%, 2009–2014, 31.8%), nor in *emm* type 28, which increased from 9.1% in 1996–2002 to 18.3% in 2003–2007 and decreased to 15.4% in 2009–2014, which is similar to results reported among adults in France [10]. Nevertheless, in other studies a re-emergence [26] or an increase [28] of *emm* type 1 has been described. However, the most prominent trend in comparison to the two previous surveillance periods in Germany is the increase of *emm* type 89 from 3.4% in 1996–2002 to 7.0% in 2003–2007 and 14.5% in 2009–2014. Comparable results have been reported from other countries [26, 28].

While our models are exploratory in nature, some underlying conditions are nevertheless clear risk factors for iGAS disease. Diabetes is a risk factor for infection with strains harbouring *spe*G. Chronic skin lesions are a risk factor for infection with strains harboring *spe*H, *spe*J, and *spe*K. Among the studied clinical complications, significant associations were found only with *spe*A 1–3 (with liver abnormality), and *emm* 3, with respiratory distress. Among the studied diagnoses, significant associations were found with *emm* type 1 (with NF), and *emm* type 28 (with septic arthritis).

The relevance of erythrogenic toxin- and superantigen genes relating to invasive infections remains inconclusive, despite extensive literature on this topic [17], particularly since they are also common in non-invasive isolates. In our study, even the statistically significant results did not result in odds ratios far above or below one. Most *emm* types were characterized by the presence of one or two specific toxin gene profiles [29, 30]. Hypothetically, at least one toxin gene is required in order for severe GAS disease to manifest [27]. Indeed, in our study, only 10 of 719 cases (1.4%) did not have any of the superantigen genes we examined, of which two cases were from patients with a co-occurring serious illness (diabetes). There are no clear statistical relationships between diagnosis or clinical complications and the samples without any detected superantigens. Samples without any of the studied superantigens were from only five *emm* types, *emm*77 (n = 5), *emm*60 (n = 2), *emm*3 (n = 1), emm63 (n = 1), and emm165 (n = 1). Since we did not examine superantigen *sme*Z, we cannot rule out the possibility that these ten samples harbour this superantigen. Further research is necessary to elucidate the interrelation between superantigen gene combinations, *emm* types and disease pattern of iGAS infections.

## Supporting information

S1 Table Diagnoses, complications, underlying conditions, *emm* types, and superantigen genes in 719 cases of invasive Group A Streptococcus disease in Germany from 2009–2014. (XLSX)
